# Supplementary material for: A Critical View of the Application of the APEX Software (Aqueous Photochemistry of Environmentally-Occurring Xenobiotics) to Predict Photoreaction Kinetics in Surface Freshwaters
Source: Molecules. 2019 Dec 18;25(1):9. doi: 10.3390/molecules25010009 (PMC7017383; doi:10.3390/molecules25010009)
Supplement: Supplementary file 1 [file molecules-25-00009-s001.zip › 3.2.4_gcc-4.4.0.zip!/3.2.4_gcc-4.4.0/3.2.4_gcc-4.4.0/doc/octave/html/interpreter/Permutation-Matrix-Functions.html]

Permutation Matrix Functions - Untitled


Previous: Diagonal Matrix Functions,
Up: Function Support


---

#### 20.3.2 Permutation Matrix Functions

inv and pinv will invert a permutation matrix, preserving its
specialness. det can be applied to a permutation matrix, efficiently
calculating the sign of the permutation (which is equal to the determinant).

A permutation matrix can also be returned from the built-in functions
lu and qr, if a pivoted factorization is requested.

The sparse function will convert a permutation matrix efficiently to a
sparse matrix.
The find function will also work efficiently with a permutation matrix,
making it possible to conveniently obtain the permutation indices.
